# Primary Pulmonary Hypoplasia With Congenital Alveolar Dysplasia Associated With *TBX4* Gene Deletion: A Case With Autopsy and Molecular Findings

**DOI:** 10.1177/10935266251322326

**Published:** 2025-02-26

**Authors:** Evelyn O. Ilori, Christine Kahlow, Rolando Garcia, Syed Ahmed, Charles Timmons, Tetyana H. Nesterenko

**Affiliations:** 1 Department of Pathology, University of Texas Southwestern Medical Center, Dallas, TX, USA; 2 Division of Neonatal-Perinatal Medicine, University of Texas Medical Center, Dallas, TX, USA

**Keywords:** pulmonary hypoplasia, TBX4, congenital alveolar dysplasia, respiratory distress, neonatal lung disease

## Abstract

Acute respiratory distress in a neonate is a potentially critical condition with multiple possible causes. Developmental etiologies are particularly problematic by virtue of being refractory to routine modalities for enhancing ventilation and oxygen exchange. Some genetic causes of neonatal respiratory distress, such as surfactant protein deficiencies and alveolar capillary dysplasia with misalignment of pulmonary veins, are well known, and sequencing panels have been formulated to detect them. We present a case of fatal neonatal respiratory insufficiency in which the autopsy showed primary pulmonary hypoplasia and congenital alveolar dysplasia. A sequencing panel of genes associated with heritable pulmonary disorders gave a normal result; however, a chromosomal microarray identified a heterozygous deletion encompassing the *TBX4* gene on chromosome 17. Haploinsufficiency for *TBX4* is a known cause of disturbed pulmonary development. This case illustrates why work-up of pulmonary developmental disorders must look beyond standard sequencing panels in some instances, if rare causes of pulmonary maldevelopment such as deletions causing haploinsufficiency are not to be missed.

## Introduction

Acute neonatal respiratory distress is a potentially critical condition with multiple causes. Developmental etiologies are particularly problematic by virtue of being refractory to routine modalities for enhancing ventilation and oxygen exchange. Some genetic causes of neonatal respiratory distress, such as surfactant protein deficiencies and alveolar capillary dysplasia with misalignment of pulmonary veins, are well known, and sequencing panels have been formulated to detect them. Heterozygous deletions may not be detected by sequencing alone, and the cause of phenotypes produced by haploinsufficiency phenotypes may go unrecognized.

## Case Report

A female neonate was delivered at 37 weeks and 1 day gestation to a 30-year-old gravida 2, para 1 (G2P1) mother at a tertiary care facility. The pregnancy was complicated by maternal anemia, and prenatal screenings were unremarkable. The mother presented with spontaneous onset of labor. Rupture of membranes occurred 32 minutes before the infant was delivered via an uncomplicated vaginal delivery with delayed cord clamping for 1 minute. At 90 seconds of life, the neonate was cyanotic, with a heart rate ranging from 70 to 90 beats per minute. About 1- and 5-minute Apgar scores were 5/5. The infant was intubated at 11 minutes of life for continued hypoxia. A 10-minute Apgar score was not assigned, as the infant was immediately transferred to the neonatal intensive care unit (NICU).

The infant was continued on positive pressure ventilation due to hypoxia and suspected congenital cardiac disease in the NICU. Initial arterial blood gas was pH 6.8, CO2 124, PaO2 10, and base excess of −15. The infant’s hypoxic-hypercarbic respiratory failure was refractory to surfactant administration, conventional and high-frequency mechanical ventilation, inhaled nitric oxide, and prostaglandin E1 administration. Hypotension was refractory to volume resuscitation and catecholamine administration. A bedside echocardiogram suggested obstructive anomalous pulmonary venous return and depressed ventricular function bilaterally.

The infant experienced 2 episodes of cardiac arrest, likely secondary to bilateral pneumothoraces, relieved by bilateral chest tube placement. The neonate’s condition continued to deteriorate, with worsening oxygen saturation, intermittent bradycardia, and persistent acidosis unresponsive to further medical interventions. After transitioning to comfort care, the infant expired at 4 hours of life.

**Figure 1.**
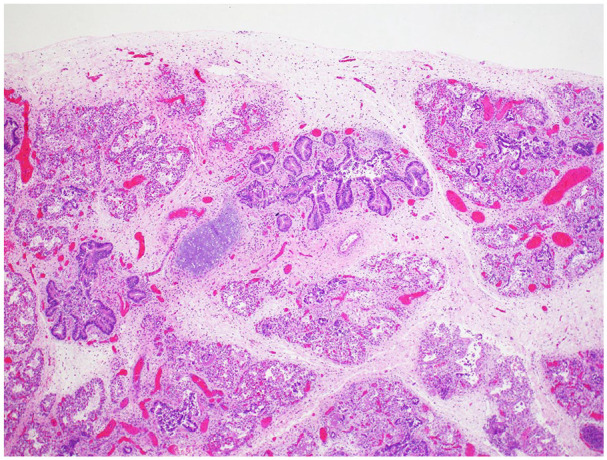
Peripheral extension of bronchioles and poorly developed lobules consistent with pulmonary hypoplasia (H&E, 4× original magnification).

**Figure 2.**
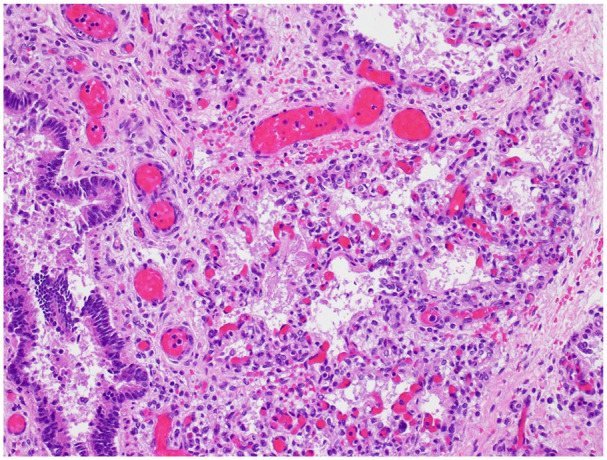
Developing airspaces show capillarization of septa and mesenchymal crests consistent with late canalicular/early saccular development (H&E, 20× original magnification).

**Figure 3.**
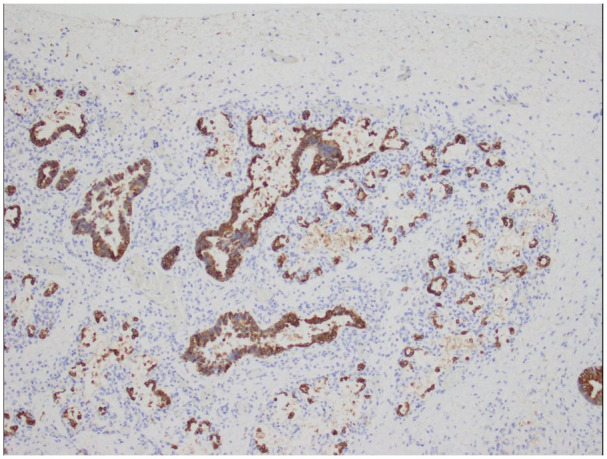
CK7 immunostaining illustrates attenuation of the columnar epithelium from the bronchioles into the developing saccules of distal airspaces (10× original magnification).

**Figure 4.**
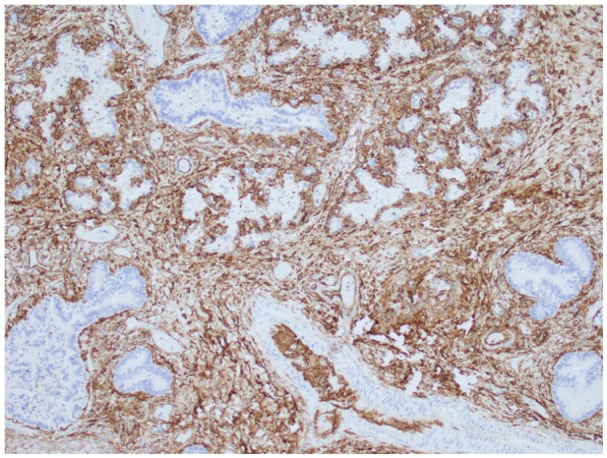
CD34 immunostaining highlights the complexity and angulation of distal airspaces and mesenchymal crests protruding into the developing airspaces (10× original magnification).

An autopsy limited to the chest and abdomen was performed. The neonate weighed 2700 g (mean for 37-week gestation = 2424 ± 535 g), with crown-rump length 33.0 cm (mean = 32.7 ± 3.2 cm), and head circumference 34.0 cm (mean = 32.5 ± 0.8 cm).^1^ The lung weight/body weight ratio was 0.009 (25.1 g/2700 g, mean = 0.0179 ± 0.0044^2^), which is considered diagnostic of pulmonary hypoplasia. Histology shows peripheral extension of bronchioles and poorly developed lobules indicative of underlying pulmonary hypoplasia (Figure 1). The radial alveolar count (RAC) was 1.0 (mean = 4.5 ± 1.74).^3^ Developing airspaces show capillarization of septa and mesenchymal crests consistent with late canalicular/early saccular development and hence congenital alveolar dysplasia for 37 weeks gestation (Figure 2). CK7 immunostaining illustrates attenuation of the columnar epithelium from the bronchioles into the developing saccules of distal airspaces (Figure 3). CD34 immunostaining highlights the complexity and angulation of distal airspaces and mesenchymal crests protruding into the developing airspaces (Figure 4). There was no evidence of alveolar capillary dysplasia with misalignment of pulmonary veins. The pulmonary venous return was normal, but the veins had very small lumens. The spleen was very small (1.8 g, mean = 8.11 ± 3.3 g). Weights for all the other organs were within expected ranges for gestational age.^1^ Peripheral blood aerobic culture and lung aerobic and anaerobic cultures were negative. Electron microscopy revealed normal surfactant lamellar bodies. Skeletal radiography was normal for age.

Cultured skin fibroblasts were sent for next generation sequencing of a panel of 124 genes associated with inherited pulmonary disorders (Greenwood Genetic Center, Greenwood, SC) and for conventional karyotyping and chromosomal microarray analysis (UT Southwestern Medical Center, Dallas, TX). The pulmonary gene sequencing panel identified no alternative etiology for the patient’s condition, including no pathogenic variants in genes coding for surfactant proteins or in *FOXF1*, responsible for alveolar capillary dysplasia with misalignment of pulmonary veins. The patient’s karyotype was normal female, 46XX, and sequencing yielded a normal result. However, microarray analysis detected a 300 kb deletion at 17q23.2 encompassing the entire *TBX2* and *TBX4* genes and the distal end of the *BCAS3* gene. This deletion would have been too small to detect in the conventional karyotype. *TBX4* was included in the sequencing panel’s normal result, indicating that the *TBX4* gene homologous to the deletion remained intact.

## Discussion

Pulmonary hypoplasia is defined by small size and underdevelopment of 1 or both lungs.^4^ Several pathological criteria have been used for the diagnosis, with the most common being the lung weight-to-body weight (LW-BW) ratio. Historically, a LW-BW ratio <0.012 is diagnostic in infants 28 weeks gestation and older, however a more recent study supported a less conservative discriminator LW-BW of 0.0179 ± 0.0044.^2,5^ The present case was hypoplastic by either criteria. RAC, as modified for the canalicular/saccular phase, was also much reduced.^3^ Potential causes include lung compression from an intra- or extra-thoracic source, oligohydramnios, abnormal or absent fetal breathing movements, environmental agents, decreased blood flow to the lungs, or genetic causes; sometimes the cause remains unknown.^6^ Pulmonary hypoplasia usually presents in newborns as severe respiratory distress and hypoxia and is often associated with pulmonary hypertension. Severe cases can be incompatible with postnatal life.^7^

This patient’s pulmonary hypoplasia was accompanied histologically by deficient alveolar development and maturation, which appeared to have arrested at the late canalicular/early saccular stage of development. This histology falls into the broader category of diffuse developmental disorders of the lung, which includes congenital acinar dysplasia, characterized by lung growth arrest in the pseudoglandular or early canalicular phase, congenital alveolar dysplasia, with arrest in the late canalicular or saccular phase, and alveolar capillary dysplasia with misalignment of pulmonary veins, with abnormal capillary, venous and arterial development in the lungs.^8^ While some variation in development within this patient’s lungs was observed, the overall pattern was most consistent with congenital alveolar dysplasia.

While the cause for respiratory failure in this case was identified anatomically, the probable underlying genetic change was identified by chromosomal microarray showing a 17q23.2 deletion that included several important genes. *TBX4* (T-box transcription factor 4) is a transcription factor critical for development of the lung and bones of the pelvis and lower extremities.^9^ Specifically, *TBX4* is expressed in the pulmonary mesenchyme of the embryo and is important in branching morphogenesis of the developing airways.^10^ Haploinsufficiency of *TBX4*, through deletion or mutational inactivation, is sufficient to cause a disturbance in lung development, sometimes along with skeletal dysmorphology.^9^ Pathogenic haploinsufficiency also means that the deletion is probably de novo and not inherited. Reduced pulmonary expression of *TBX4* or any of its target genes causes impaired branching, leading to congenital acinar or alveolar dysplasia.^11^ *TBX4* mutations and deletions can be associated with skeletal abnormalities of the feet and the pelvis.^12^ Homozygous mutations of the *TBX4* gene have been associated with posterior amelia with pelvis and pulmonary hypoplasia syndrome (PAPPAS), while heterozygotes for the mutations may exhibit the milder ischio-coxo-podo-patellar (“small patella”) syndrome with or without pulmonary arterial hypertension.^13^ In the present case, the feet were normal, and the reported pelvic bone abnormalities cannot be diagnosed until several years beyond infancy when ossification is more advanced.

*TBX2* has been implicated in development of the heart, limbs, craniofacial structures, and tumorigenesis.^14^ and the role of *TBX2* in lung development has been demonstrated in mouse models.^15^ Human lung maldevelopment independent of *TBX4* has not yet been described, but deletion of *TBX2* might be a contributing factor along with *TBX4. BACS3* is associated with embryogenesis and tumor angiogenesis in humans, and loss-of-function variants are associated with an autosomal recessive neurodevelopmental disorder. *BACS3* is thus unlikely to have played a major role in this case.^16^

While the pulmonary veins had normal connections to the heart and lungs, they were small, possibly secondary to the pulmonary hypoplasia or as a feature of the primary genetic alteration. An association of severely hypoplastic spleen with this genetic deletion has not been reported previously.

Although rare, *TBX4*-mediated pulmonary hypoplasia with acinar or alveolar dysplasia is an important diagnosis to consider in newborns with unexplained respiratory distress. This is especially important to consider in newborns, without apparent characteristic skeletal abnormalities. The present case illustrates the limitations of sequence analysis alone for pathogenic variants detection and suggests that microarray analysis should be considered as part of a comprehensive work-up.
